# Observation and execution of upper-limb movements as a tool for rehabilitation of motor deficits in paretic stroke patients: protocol of a randomized clinical trial

**DOI:** 10.1186/1471-2377-12-42

**Published:** 2012-06-18

**Authors:** Denis Ertelt, Claudia Hemmelmann, Christian Dettmers, Andreas Ziegler, Ferdinand Binkofski

**Affiliations:** 1 Center for Clinical Trials, University of Lübeck, Maria-Goeppert-Straße 1, 23562, Lübeck, Germany; 2 Institute of Medical Biometry and Statistics, University of Lübeck, University Hospital Schleswig-Holstein - Campus Lübeck, Maria-Goeppert-Straße 1, 23562, Lübeck, Germany; 3 Kliniken Schmieder Konstanz, Eichhornstraße 68, 78464, Constance, Germany; 4 Cognitive Neurology, Department of Neurology, RWTH Aachen University Hospital, Pauwelsstraße 30, 52074, Aachen, Germany

## Abstract

**Background:**

Evidence exist that motor observation activates the same cortical motor areas that are involved in the performance of the observed actions. The so called “mirror neuron system” has been proposed to be responsible for this phenomenon. We employ this neural system and its capability to re-enact stored motor representations as a tool for rehabilitating motor control. In our new neurorehabilitative schema (videotherapy) we combine observation of daily actions with concomitant physical training of the observed actions focusing on the upper limbs. Following a pilot study in chronic patients in an ambulatory setting, we currently designed a new multicenter clinical study dedicated to patients in the sub-acute state after stroke using a home-based self-induced training. Within our protocol we assess 1) the capability of action observation to elicit rehabilitational effects in the motor system, and 2) the capacity of this schema to be performed by patients without assistance from a physiotherapist. The results of this study would be of high health and economical relevance.

**Methods/design:**

A controlled, randomized, multicenter, paralleled, 6 month follow-up study will be conducted on three groups of patients: one group will be given the experimental treatment whereas the other two will participate in control treatments. All patients will undergo their usual rehabilitative treatment beside participation in the study. The experimental condition consists in the observation and immediate imitation of common daily hand and arm actions. The two parallel control groups are a placebo group and a group receiving usual rehabilitation without any trial-related treatment. Trial randomization is provided via external data management. The primary efficacy endpoint is the improvement of the experimental group in a standardized motor function test (Wolf Motor Function Test) relative to control groups. Further assessments refer to subjective and qualitative rehabilitational scores. This study has been reviewed and approved by the ethics committee of Aachen University.

**Discussion:**

This therapy provides an extension of therapeutic procedures for recovery after stroke and emphasizes the importance of action perception in neurorehabilitation The results of the study could become implemented into the wide physiotherapeutic practice, for example as an ad on and individualized therapy.

## Background

Studies on primates and a growing number of electrophysiological and imaging studies in humans provided evidence that observation–dependent activation of motor areas is mediated by the so called “mirror neurons” [1-3]. These neurons discharge both when a goal-directed action is performed and when the same or a similar action is observed being performed by another human [1,2]. Action observation is supposed to induce a re-enactment of similar actions stored in human brains [4-6] possibly by inducing simulation of the ongoing actions [7]. It is likely that action observation leads to organisational changes in the brain [4] and may participate via the mirror neuron system (MNS) in learning of motor skills [8]. Concluding from these findings, we assumed that the MNS has the capacity to stimulate the cerebral motor areas by the observation of external movements. Physiological studies clearly show that observation of congruent actions significantly facilitates learning of basic movements in stroke patients and that movement execution alone or movements execution combined with observation of non-congruent actions have no such effect [9].

Basing on these results we conducted a pilot study, in which we established a novel physiotherapeutic scheme, the so called "videotherapy", combining action observation with action execution for rehabilitation of motor deficits after stroke [10-13]. We arranged our therapeutic scheme in a way that it would allow the most possible unhindered activation of the MNS by focused action observation. Further on, we included physical exercises to strengthen any activation effects on the motor areas thus permitting and reinforcing cerebral reorganization. Our study was designed to test for the direct top-down stimulation effect of motor observation on the treatment of chronic motor arm deficit after stroke. Based on the previous experiences using mental techniques in neurorehabilitation [14] we combined action observation with the direct effects of the top-down action execution. Our hypothesis was that the activation of motor areas by action observation becomes reinforced by the concomitant execution of the observed actions [15]. Therefore, action observation, along with reinforcement by actual action execution, should be a powerful tool in neurorehabilitation. The results of this pilot study [10-13] confirmed this prediction. In this study we used a design with one treatment (n = 8) and one control group of post stroke patients (n = 8) in a chronic stage with mild to moderate arm paresis. The experimental condition consisted in watching video clips containing daily activities and in imitating these activities with the paretic limb directly afterwards. The control condition matched the treatment condition, except for watching slideshows of geometric symbols instead of movements. The main statistical analysis showed a highly significant improvement in all experimental group members during the course of treatment as evident from the objective (Wolf Motor Function Test WMFT [16]: p = 0.009; Frenchay Arm Test FAT [17]: p = 0.007; Wilcoxon-Rank-Sum-Test) and the subjective (Stroke Impairment Scale SIS [18]: p = 0.013) scales. The control group of patients did not show noticeable improvement during the course of the training (WMFT: p = 0.288; FAT: p = 0.159; SIS: p = 0.131; Wilcoxon-Rank-Sum-Test). The direct comparison between the treatment and control groups confirmed that a better improvement of the motor skills was acquired in the treatment group compared to the control group (WMFT: p < 0.05; FAT: p < 0.001; SIS: p < 0.003; Mann–Whitney-*U*-Test). Additionally, the effects of action observation therapy on the reorganization of the motor system were investigated by functional magnetic resonance imaging (fMRI) using an independent sensorimotor task containing object manipulation [10-13]. The major result of the fMRI study was revealed by direct comparison of the changes in neural activation during the course of the treatment between the experimental and the control groups. This contrast yielded a significant rise in activation in the bilateral ventral premotor cortex, bilateral superior temporal gyrus, the supplementary motor area (SMA) and the contralateral supramarginal gyrus. Our results suggest that action observation, by reactivating those motor areas which contain the action observation-execution matching system, has a positive additional impact on recovery of motor functions after stroke.

Furthermore, recent publications stated that only the non-affected hemisphere could be stimulated by mental techniques, like motor imagery [19,20]. These studies further described that mental techniques facilitate recovery only in left-hemispheric strokes, and not in right-hemispheric strokes. To investigate this issue we tested stroke patients using fMRI and the aforementioned paradigm of action observation [11]. As opposite to these results, our own data obtained from eight right-hemispheric and eight left-hemispheric stroke patients revealed that both the affected as well as the non-affected hemisphere were activated by action observation [21,22]. This result represents a further important prerequisite for application of the videotherapy [23].

In sum, in the field of stroke rehabilitation, the videotherapy represents a treatment of motor impairments based on a clear physiological principle - the mirror neuron system. The ambulatory clinical pilot and our further studies on patient dedicated interventions let us assume the feasibility and effectiveness of a training session using action observation and physical imitation.

The planned trial will examine the following hypotheses: 1. for stroke patients in sub-acute state, observation of goal-directed actions in combination with motor performance ("videotherapy") has a greater neurorehabilitative effect than standard rehabilitation techniques alone, 2. Videotherapy has the advantage to be easily administered at patient’s home who will perform it on her/his own and without assistance of a physiotherapist.

## Methods/design

### Trial design

The form of our clinical study will be: multicenter, open, randomized, placebo-controlled, paralleled group trial with three investigative arms conducted in Germany (5 sites). This study aims at demonstrating superiority of the videotherapy over both physical training and standardized physical therapeutic treatments. We will use equal number of patients for the randomization on the three groups (1:1:1).

### Participants

In general, eligible participants will be all adults aged 30 or over with first clinically evident stroke, resulting in primarily mild to moderate unilateral upper limb paresis. Patients with more severe motor symptoms or further medical or neuropsychological symptoms will not be eligible to participate. See Table 1 for detailed inclusion and exclusion criteria.

**Table 1:** Inclusion and exclusion criteria

**Inclusion criteria**	
Patients that are both psychically and physically eligible, with good general state of health and nutritional condition for experimental treatment both in the intensity and duration planned in the presented trial (decided by the investigator).	
Hospitalized patient: discharge from rehabilitation within the following seven days (patient's data in the respective clinic)	
Insult at least 4 weeks in the past, hemorrhagic stroke at least 6 month in the past (controlled via medical history, patient's data in the respective clinic).	
First clinically evident stroke so that the patient has no history of stroke related trainings and treatments (controlled via medical history, patient's data in the respective clinic, and note from the general practitioner from outpatients, respectively).	
Ischemic cortical or subcortical lesions in middle cerebral artery territory or brain stem infarction resulting mainly in defined motor impairments (controlled via medical history, patient's data in the respective clinic, and note from the general practitioner from outpatients, respectively).	
Primarily motor symptoms with also primarily unilateral upper limb paresis (controlled by standard neurological examination).	
Minimal movement ability of the paretic limb (controlled by MRC index ≥2 and ≤4: hand extension against gravity at wrist = 20° and at metacarpophalangeal and interphalangeal joints of each of the fingers = 10°) to participate in the treatments’ physical training tasks.	
If any, only minimal aphasic symptoms allowing for understanding and following instructions during test administration and treatment (controlled by administration of the Token test ≤ 21 incorrect reactions).	
If medication is needed: concomitant medication with effects on the motor system, vigilance and/or cardiovascular system will be kept unchanged throughout of the participation in the study (controlled via discharge letter from hospitalized patients, and note from the general practitioner from outpatients, respectively; as well as by questioning the patient during the weekly telephone calls and the assessments throughout the training phase).	
Signed informed consent to participate in the trial.	
EU-citizen for legal aspects of participation.	
A DVD-Player is either in possession of the participant or is lent to him by relatives or friends to allow participant to conduct the treatment at home.	
**Exclusion criteria**	
Impaired level of consciousness that could prevent patient to understand and follow instructions throughout the intervention, and further result in inability to hold attentiveness and concentration to the treatment (controlled via standard neurological examination, subjective impression of the investigator during administration of test inventory – i.e. patient is unable to understand and/or follow instructions).	
Severe untreated psychiatric disorder, severe pulmonary or cardiovascular disease, or epilepsy that could lead to reduced abilities to participate in the treatments’ task (controlled via discharge letter from hospitalized patients, and note from the general practitioner from outpatients, respectively). If any of the referred diseases is treated with allowed stable medication, patient may not be excluded from study participation.	
Severe joint deformity of arthritic origin or chronic pain that could reduce significantly the patient's abilities to perform tasks that demand a functional physical execution, thus resulting in the masking of expected training effects (controlled via standard neurological examination).	
Motor problems not primarily unilateral or excessive pain in major affected limb that could reduce the patient's abilities in tasks requiring a functional physical execution, thus resulting in the masking of expected training effects (controlled via standard neurological examination).	
Dementia symptoms that could prevail over the administration of test inventories and treatment (controlled by administration of the Mini-Mental-State Examination, MMSE, score ≤ 22).	
Depressive symptoms that could result in major difficulty of the patient's motivational compliance to follow instructions and to participate in the interventions’ tasks throughout the treatment (controlled by administration of the Beck Depression Inventory, BDI, score ≥ 18).	
Apraxic symptoms that could lead to impaired abilities to follow instructions (controlled by administration of the Florida Apraxia Screening Test, FAST, ≤ 9 correct reactions).	
Neglect symptoms that could lead to impaired abilities to participate in the treatments observational tasks (controlled by administration of the Albert’s Neglect Test, ANT, minimal 2 lines unchecked).	
Actual neuroleptic treatment for psychiatric reasons; no constant concomitant medication (controlled via discharge letter from hospitalized patients, and note from the general practitioner from outpatients, respectively).	
Planned, but not actual treatment with psychotropic and/or antiepileptic medication or, if already in treatment, change of dose of the referred medications during the phase between the screening and post-trial assessments (controlled via discharge letter from hospitalized patients, and note from the general practitioner from outpatients, respectively).	
Planned start of other rehabilitation therapies that might interfere with the trial treatment in the next eight weeks from time point of recruitment.	
Insufficient knowledge of German language to understand and fill in the questionnaires (clinical judgment during standard neurological examination).	
Residence more than 300 kilometers from participating center that would exacerbate the regular visits of the patient in the respective center (controlled by questioning of the patient).	
Persons who are accommodated in an institution by court or administrative order (controlled by questioning of the patient).	
Any other medically untreated illness or medical treatment or drug or narcotics misuse that could interfere with the assessment of the safety, tolerability and efficacy, e.g. current bone fractures of the stroke affected limb (controlled via discharge letter from hospitalized patients, and note from the general practitioner from outpatients, respectively).	
Simultaneous participation in another (clinical) trial or interfering examination or participation in a study within 90 days prior to screening.	
People who are in a dependency/employment for the sponsor or investigator (controlled by questioning of the patient).	
Biological age <30.	
Pregnancy or suspected pregnancy. Lack of safe contraceptive measures.	

The multicenter study will take place at 5 sites in Germany: Department of Neurology of University of Leipzig, Department of Neurology of Brandenburg Klinik Bernau near Berlin, Clinic for Neurology of at Campus Lübeck University Medical Center Schleswig-Holstein, St. Mauritius Therapieklinik at Meerbusch near Düsseldorf, and the Neurological Centre of Segeberger Kliniken GmbH at Bad Segeberg. All sites represent the main venues for stroke patients and stroke-related rehabilitation in their surrounding areas, thus able to reach the expected sample size of stroke patients. Because the study centers at Lübeck and Leipzig do not provide treatment for patients in their post-stroke rehabilitation phase, these sites will recruit patients from nearby rehabilitation centers to where their sub-acute patients have been referred further on. Recruited patients therefore will have been pre-screened at the respective site and will be invited at the end of their rehabilitative treatment to the trial site for participation. All recruitment formalities and ambulatory or stationary assessments will be conducted in the sites stated above with patients transported to the respective clinics.

Patients' data will be assessed in the respective sites. Data management and monitoring of the trial sites will be conducted by the Center for Clinical Trials, University of Lübeck. Biostatistical analysis and randomization will be performed by the Institute of Medical Biometry and Statistics, University of Lübeck. An independent Data and Safety Monitoring Board (DSMB) of two clinicians and a biostatistician will to supervise patients' safety, as well as statistical analysis and interpretation.

### Interventions

One experimental and two control groups will be built. All patients of all groups will conduct their individual rehabilitational programme as prescribed by their respective neurologist beside participation in our scheme. The standard rehabilitational programs will not be limited in their amount to allow participation to our study but must not be experimental or part of another clinical trial.

The respective intervention starts with a practice at the site during the last week at their inpatient stay. Patients will be asked to start immediately with the self-administered treatment after their return back home. All patients will keep record of their training in a pre-printed diary and will receive a weekly phone call in which compliance of the patients will be monitored.

#### Experimental condition (abbr. "videotherapy")

The experimental treatment in condition videotherapy will consist of daily focused observation of actions and subsequent practice of these actions for 6 weeks with therapeutic session on each working day lasting 90 minutes. The treatment material will consist of 1 DVD containing a set of video clips for each patient. These video clips will show dedicated hand and arm actions with household objects, each presented from different perspectives and each lasting for 6 minutes. The total duration of the treatment program will be 90 minutes per working day, the patient will be informed by a manual each day which set of films he/she has to watch. After watching each single video clip, the patient will be asked automatically by the DVD program to imitate the observed action using his/her own household objects with his/her paretic hand.

Our therapeutic scheme (videotherapy) was invented to rule out most confounding variables of the training. More precisely, 1) the therapy should elicit the most possible unhindered activation of the MNS and 2) each patient should as far as possible benefit from the therapeutic setting.

To achieve this goal we implemented the following features to our treatment:

1. Observation of well-known, non-complex hand and arm actions related to small common household objects, performed by one hand, will be the key feature of the therapy. Attentive watching of well-known actions will allow the patient-observer to comprehend their content and take details of those actions. The high familiarity and recall level of the observed actions, which belong to an average motor behavioral repertoire, allows the activation of predefined movement patterns in the patient's motor system. This should be the prerequisite for an internal simulation of the observed actions, which refers to a covert rehearsal of motor actions and is believed to ameliorate the subsequent overt action performance. We assume that this will affect the capability to induce rehabilitation effects by stimulating cerebral reorganization, as demonstrated in our pilot studies [10-13]. The video clips for the current study are mainly derived from an already evaluated pool that has been used in our pilot studies [10-13]. An assessment of the films' presented movement complexity allowed creating sequences of video clips with alternating difficulty (easy – difficult) for every day of training. Therefore, highly and moderate affected stroke patients will be able to perform at least some of the actions without getting over- or underchallenged.

2. To provide comparability of results and to avoid variability in the amount of observed actions we decided to use standardized video clips of object related movements as treatment material. Video clips will be presented from a DVD disk, a user-friendly medium that can be copied in virtually infinite quantities. Further on, DVD players are common and can be found in almost every patient's household.

3. The therapeutic schema of the videotherapy will consist of observation and consecutive training of the observed actions. We assume that the activation of the MNS by action observation should be reinforced by the consecutive execution of the observed actions. This combination should finally elicit effects of plasticity in the motor system. Our treatment schema is based on this effect: After the end of each video clip, the patient will be asked to imitate for 6 minutes the actions seen before using similar objects from his or her own household. Only the affected arm and affected hand will be used. The patient will be informed during watching of the DVD films, when to start and when to stop the training and when to take a break.

#### Control condition I (abbr. "non-video")

The treatment material for the control condition non-video will be equivalent to the experimental treatment, except for the fact that slideshows of geometric symbols will be shown instead of actions. These slideshows have been proven not to elicit any activity in the motor areas [10-13] and were therefore chosen as placebo treatment.

#### Control condition II (abbr. "standard")

No specific experimental treatment is intended for the standard-condition. However, this group's patients will receive the same schedule of assessments as the other groups and will participate in their standard rehabilitational treatments.

Further on, the patients of the non-video and the standard group will receive (additionally) the same treatment disk after the end of the individual training phase. In this, all patients, independent from their group allocation, will have the same treatment material that is believed to be effective for stroke rehabilitation. This is 1) to allow all patients to benefit from the therapy positively tested in the several pilot studies [10-13], and 2) to maintain compliance of the patients, even for being in the standard group and not receiving in the first 6 weeks of participation a special treatment.

#### Risks associated with the participation in the clinical trial

There are no known risks associated with the videotherapy or any of the two control conditions. Due to the self-administration of the treatment in dose, place, time *etc.* the individual patient has the potential possibility to regulate the treatments properties for his or her own convenience. Further on, our pilot studies (10–13) did not indicate any risks in the treatment, its materials or in its conduction. The same is true for the used control conditions.

### Outcomes

All outcome measures and assessment time points for patients are listed in Table 2.

**Table 2:** Outcome measures, assessments, and frequency and scope of study visits

**Visit**	**Screening**	**Instruction** **(Videotherapy and Non-Video Group)**	**Baseline**	**Training phase**	**End of Training**	**Follow-up**
**Time**	Week 1: Day 1-3	Week 1: Day 4-5	Week 2	Week 2 – 7 + 4 days	Week 8 + 4 days	Week 31 + 1 week
**Screening, supervision**						
**Informed consent**	X					
**Inclusion/exclusion criteria**	X					
**Background medical history**	X					
**Concomitant medication**	X					
**Screening**	X					
**Beck Depression Inventory**	X		X	X (week 5)	X	X
**Amount of training with videotherapy DVDs**						X
**Preparation of patient**						
**Instruction and training**		X				
**Primary efficacy endpoint**						
**Wolf Motor Function Test**			X		X	X
**Secondary efficacy endpoint**						
**Frenchay Arm Test**			X		X	X
**Stroke Impact Scale**			X		X	X
**Barthel-Index**	X		X	X (week 5)	X	X
**Modified Rankin Scale**	X		X		X	X
**Motor Activity Log**			X	X (week 5)	X	X
**Box and Block Test**			X		X	X
**Further treatment**						
**Telephone calls**				Weekly		
**Diary**				Daily by the patient		

#### Primary endpoint

The primary efficacy endpoint is the change in scores of the WMFT [16] between baseline and after six weeks of rehabilitation treatment between the videotherapy group, the standard and videotherapy group and the non-video group.

#### Secondary endpoints

The secondary endpoints concern quality of life related to improvement in motor function indices (tested via FAT, 17) and autonomy (tested via SIS [18], Barthel-Index - BI [24], Modified Rankin Scale - MRS [25], Motor Activity Log – MAL [26], Box and Block Test - BBT [27]). A secondary endpoint is the change in the WMFT [16] scores between baseline and after end of the treatment compared to the non-video and standard groups. The changes in the scores and mortality between all groups at all time points will be tested pairwise. Furthermore, the difference between the end of treatment and 6 month after the training will be investigated within each group.

#### Subgroup analysis

We will perform pairwise comparisons between all groups (videotherapy, non-video and standard) in dependency of the following variables: center, age, gender, race, dominance of the affected hemisphere, affected hemisphere, type and amount of additional physio- and/or occupational or other therapeutic treatments during training phase, chronicity/sub-chronicity of stroke symptoms. Chronicity is defined by manifestation of stroke related motor symptoms ≥ 6 months after the incident.

#### Further assessments

Besides the assessment of the outcome measures as listed above, several additional assessments will be conducted for screening reasons (Background medical history, Intake/prescription of concomitant medications, standard neurological examination, Florida Apraxia Screening Test - FAST [28], Albert's Neglect Test - ANT [29], Mini Mental Status Examination - MMSE [30], Edinburgh Handedness Inventory - EHI [31], Beck Depression Inventory - BDI [32], Modified Medical Research Council Scale for measuring hand muscles - MRC-Index [33]). The BDI [32] will be conducted throughout all assessment time points due to the fact that depressive symptoms can evolve in all phases of the study and can therefore prevent an unhindered conduction of the training.

### Sample size

The calculation of the sample size is based on the aim to detect a difference of 2.5 points in the WMFT [16] between the videotherapy- and the standard group concerning the change from baseline until the end of rehabilitation, corresponding to δ = 0.5, with a power of 90% in the final of two analyses. The critical difference of 2.5 points in the WMFT [16] results from our pilot study [10-13]. Allowing for a maximal drop out of 20% of the patients, 125 patients need to be included in the study for each group, rendering a total sample size of 375 (α_2_(1) = 0.035, Mann–Whitney test).

The interim analysis is conducted according to Wang and Tsiatis [34], after half of the patients in each group (63 in each group, a total of 188) have concluded the rehabilitation program. If the effect is considerably greater than assumed (δ = 0.7, corresponding to a difference of 3.5 change points), there is a probability of 80% to detect this at this early stage and hence to terminate the study early.

### Randomization: sequence generation

According to Nordle and Brantmark [35], a self-adjusting randomization plan with an allocation probability of 0.9 is utilized with stratification for center as the more important and the MRC-Index (reached scores 2, 3, and 4) [33] as the less important factor.

### Randomization: implementation

The allocation of patients will be conducted by an investigator without any clinical involvement in the trial using proper random sequences unbeknown to the patients or study team: The randomization and further allocation is performed centrally via fax at the Institute of Medical Biometry and Statistics at the University Hospital Schleswig-Holstein, Campus Lübeck, Germany (IMBS) after the respective investigator had screened the respective patient and obtained his/her written informed consent.

### Blinding

Participants are to be enrolled by the respective trial sites' personal.

Participating patients can only be blinded for the comparison of the videotherapy versus the non-video condition, but not for the comparison with the standard rehabilitation. Hence, comparing videotherapy with standard might be biased by the knowledge of the patients to receive a novel treatment. In addition, the intensity of rehabilitation will be comparable between the videotherapy and the non-video group, but not necessarily between both motor exercise groups and the standard group. Therefore, a difference between videotherapy and standard might be caused by a more intense training in the first group. These possible influences will have to be considered in the interpretation of possible study outcomes.

### Statistical analysis

#### Study population

According to the "ICH Topic E 9" there are two different sets (intention to treat set and per protocol set) for the statistical analysis.

The primary analysis will be based on the intention to treat population where missing values will be imputed with a mean imputation. Similar rates of missing baseline observations between the treatment groups as well as analogous results from sensitivity analyses will be used to support the interpretation. As sensitivity analyses, the imputation methods "Last Observation Carried Forward" principle and best or worst case imputation will be applied.

A secondary analysis will be based on the per protocol population. Here we consider only patients which complete at least 66,67% of the training sessions. This value is derived from the pilot study [10], where significant treatment effects occurred after duration of 20 training days. The analyses will be the same as with the intention to treat.

#### Primary endpoint

The score difference cannot be assumed to be normally distributed, so that distributions will be compared by analyzing the probability that an observed change score X_Videotherapy_ in the videotherapy population is greater than an observation in the non-video X_Non-video_ or the standard population X_Standard_:

H01:·PXVideotherapy>XStandard=12versusH11:·P(XVideotherapy>XStandard)≠12

and

H02:·PXVideotherapy>XNon−video=12versusH12:·P(XVideotherapy>XNon−video)≠12

The comparisons will be performed in a hierarchical manner according to a suggestion by Wiens [36,37]. For this procedure, the total significance level α is split into one portion for the first hypothesis α(1) and one for the second hypothesis α(2) = α – α(1). Accordingly, as we expect a major difference between the novel video-based rehabilitation and the standard physiotherapeutic rehabilitation, this difference will be tested first at the significance level α(1). If the null hypothesis can be rejected, we will secondly test the difference between videotherapy and non-video at the full significance level α. However, if the first hypothesis cannot be rejected, the second hypothesis can still be tested at the significance level α(2). The hypotheses will be tested using the exact *U*-test according to Mann and Whitney, and the specific significance levels are fixed using a sequential analysis plan (see Table 3).

**Table 3:** Procedure of statistical analysis

**Analysis i**	**Time point**	**Hypothesis**	**Nominal level α**_**i**_	**Detectable effect δ**_**i**_	**Power**
1	Interim analysis	Video vs. standard	0.01	0.7	80%
		Video vs. non-video	0.01	0.7	80%
2	Final analysis	Video vs. standard	0.035	0.5	90%
		Video vs. non-video	0.01 or 0.045	0.47 or 0.55	90%

#### Secondary endpoints

The analysis of the secondary endpoints will be performed exploratory. The hypothesis

H03:·PXNon−Video>XStandard=12versusH12:·PXNon−video>XStandard≠12

will be tested using the exact *U*-test according to Mann and Whitney at the significance level α = 0.05. The change scores of the BBT, MAL, SIS, FAT, MRS, and BI as well as the WMFT and mortality between groups at all time points will be tested pairwise using the exact *U*-test according to Mann and Whitney and Chi-squared test respectively at the significance level α = 0.05. The effect measures will be the difference of medians with 95% confidence interval (Hodges-Lehmann) and the difference of proportions with 95% confidence interval (independent samples), respectively. Furthermore, the difference between end of training and 6 month after the training will be investigated within each treatment group concerning the BBT, MAL, SIS, FAT, MRS, and the BI as well as the WMFT scores. Therefore, 95% confidence intervals are computed for these differences (paired samples).

#### Subgroups

In the subgroup analyses the results of the test inventories and assessments stated above are used.

#### Interim analyses

After half of the patients have concluded the rehabilitation program, an interim analysis will be performed. This will allow for an early termination of the study if the effects are greater than assumed for sample size determination while keeping an overall significance level of α = 0.05. The interim analysis is planned according to the designs suggested by Wang and Tsiatis [34] and yields a nominal significance level of α1 = 0.01 for the interim analysis. At this time point, all three null hypotheses regarding the primary endpoint as stated above are tested at the full nominal significance level, and the study is terminated only if all null hypotheses are rejected.

For the final analysis, the three hypotheses of the primary endpoint are tested at a nominal significance level of α2 = 0.045 in the hierarchical manner described above. Specifically, the first hypothesis is tested at a significance level of α2(1) = 0.035. If this null hypothesis is not rejected, the second null hypothesis is tested at the remaining significance level of α2(2) = 0.01. However, if the first null hypothesis is rejected, the second is tested at the full nominal significance level of α2 = 0.045.

This procedure renders the following effects detectable in the different stages, as presented in Table 3. The effects are measured by the differences of medians, for which 95% confidence intervals (Hodges-Lehmann) are computed for both group comparisons.

#### Criteria for termination of the study

The study can be terminated prematurely if circumstances become known that suggest a significant deterioration in the expected benefit-to-risk ratio of either study arm in comparison to the other. The DSMB will monitor any safety issues of the trial and will make a recommendation based on their evaluation.

In the event of substantial and irreparable deficiencies in data quality, inadequate compliance, or deficient patient recruitment, the study can be terminated prematurely: The DSMB will monitor these issues to make a recommendation for a premature termination, if a conclusion regarding the study question can no longer be expected to be reached on the basis of the data.

Further on, an early termination of the study will be made, if the statistical effects for testing the statistical hypotheses are greater than assumed for sample size determination; these effects will be calculated by the planned interim statistical analysis.

### Ethical approval and registration

This study has been reviewed and approved by the medical ethics committee of the RWTH Aachen university, Aachen, Germany (reference no.: 106/10) and has been registered at ClinicalTrials.gov (identifier: NCT01242956) Patients receive verbal and written information about the study and written informed consent will be obtained before randomization.

## Discussion

The proposed treatment, based on the neurophysiological principle of the mirror neuron system, provides a new and relevant extension of therapeutic procedures for recovery after stroke. The results of the planned multicenter study might be implemented to the standard concepts of physiotherapeutic practice, for example as an add-on therapy. Therefore, the results of our study should be of great interest for the community of neurologists and physiotherapists and most relevant for health economy.

The planned multicenter project will introduce novel aspects to the rehabilitation of stroke. First, focused observation of goal-directed actions before their execution constitutes a novel aspect in the treatment of motor deficits after stroke. Action observation activates central representations of actions through the mirror neurons system. The subsequent repetitive execution of the observed actions reinforces the cortical representation of action. The mirror neuron system provides a clearly defined neurophysiologic background for this new approach. Second, the new treatment is aimed at patients in the sub-acute state after stroke. However, it should also be beneficial for severely handicapped patients in the acute state, when applied prior to physiotherapy. Therefore, because activation of action representations by observation takes place also without the performance of active movements, this new therapy is also potentially suitable for patients with acute strokes. Thirdly, the videotherapy can be focused on the individual pattern of motor impairments in the future. In times of restricted resources in the health system, it is of paramount importance to develop cost-efficient rehabilitation programs.

Additionally, one of the major goals of this proposal is to implement a home-based practice program for patients and to evaluate its effectiveness. Another potential advantage of this home-based treatment is that patients are educated to carry responsibility for their future well-being. They are trained to perform exercises on their own. This is in line with current trends in the health system to treat the patient as responsible partner able for shared decision making. Up to date there exists no rationale for selecting the appropriate physiotherapeutic regime in a particular individual patient. Improving our understanding, how and where different therapeutic strategies work or act in the brain, will optimize the rational basis for applying different therapies.

## Abbreviations

ANT: Albert’s Neglect Test; BBT: Box and Block Test; BDI: Beck Depression Inventory; BI: Barthel-Index; CRF: Case report form; DVD: Digital versatile Disc; DSMB: Data and Safety Monitoring Board; EHI: Edinburgh Handedness Inventory; FAST: Florida Apraxia Screening Test; FAT: Frenchay Arm Test; FMRI: Functional magnetic resonance imaging; ICH: International Conference on Harmonization; IMBS: Institute of Medical Biometry and Statistics; LOCF: Last Observation Carried Forward; MAL: Motor Activity Log; MMSE: Mini-Mental-State Examination; MNS: Mirror neuron system; MRC: Medical research council; MRS: Modified Rankin Scale; PID: Patient identification number; RWTH: Rheinisch-Westfälische Technische Hochschule; SIS: Stroke Impairment Scale; SMA; Supplementary motor area; SOP: Standard operating procedure; WMFT: Wolf Motor Function Test.

## Competing interests

The authors declare that they have no competing interests.

## Authors’ contributions

FB is the coordinating investigator. DE is project manager and responsible for drafting the manuscript. AZ is the trail statistician. DE and FB designed the study and obtained funding for the study. AZ and CH provided the statistical considerations of the study design and the evaluation plan of the trial. CD assisted in scientific research and treatment design. Monitoring and data management are provided by the Center for Clinical Trials, University of Lübeck. All authors have read and approved the final manuscript.

## Pre-publication history

The pre-publication history for this paper can be accessed here:

http://www.biomedcentral.com/1471-2377/12/42/prepub
